# The roles of secretory autophagy in mitochondria release via extracellular vesicles: waste disposal and food delivery?

**DOI:** 10.3389/fcell.2024.1392810

**Published:** 2024-05-24

**Authors:** Yuhan Gong, Yucheng Zhou, Linhui Feng, Yuting Zhao

**Affiliations:** ^1^ Institute of Future Agriculture, Northwest A&F University, Yangling, China; ^2^ College of Veterinary Medicine, Northwest A&F University, Yangling, China; ^3^ College of Life Sciences, Northwest A&F University, Yangling, China

**Keywords:** mitochondria, secretory autophagy, extracellular vesicles, mitophagosome, membrane fusion, mitophagy, autophagosome formation

## Introduction

Autophagic degradation of mitochondria, or mitophagy, has been widely studied as an important process for mitochondrial quality control (see review (Picca et al., 2023)). Intriguingly, recent studies reveal that mitochondrial clearance relies on extracellular vesicles (EVs) in the absence of the mATG8-conjugation system (Tan et al., 2022) or functional lysosomes (Liang et al., 2023), indicating the emerging role of secretory autophagy in organelle secretion. Moreover, mitochondria can be packed into newly identified types of EVs, such as cardiac exophers during heterophagy for damaged mitochondria release from cardiomyocytes (Nicolas-Avila et al., 2020; Nicolas-Avila et al., 2022), mitosomes (specialized migrasomes) during mitocytosis for damaged mitochondria release from migrating cells (Jiao et al., 2021), and mitophers during mitopherogenesis for healthy mitochondria release from worm spermatids (Liu et al., 2023). Mitochondria-containing EVs (hereinafter referred as to Mito-EVs) can later be internalized by macrophages for elimination (Nicolas-Avila et al., 2020; Liang et al., 2023) or stimulate innate immune response in other recipient cells (Tan et al., 2022).

In this opinion piece, we compare the molecular mechanisms underlying the formation of five types of recently reported Mito-EVs and discuss the potential roles of secretory autophagy in releasing both damaged and healthy mitochondria.

### Mitochondria release via extracellular vesicles

Mitochondria have been reported to transfer across cells through tunneling nanotubes, within extracellular vesicles, or in a carrier-free manner (see review (Borcherding and Brestoff, 2023)). In the past two decades, mitochondrial-derived vesicles (MDVs) containing mitochondrial components and Mito-EVs containing fragmented or intact mitochondria have been observed in many cell types like mesenchymal stem cells (Spees et al., 2006; Islam et al., 2012), endothelial cells (Tramontano et al., 2010), platelets (Boudreau et al., 2014), and iPSC-derived cardiomyocytes (Ikeda et al., 2021). MDVs and Mito-EVs are formed under physiological and pathological conditions and may exert therapeutic effects in various diseases (see review (Zhou et al., 2023; Hazan Ben-Menachem et al., 2024)). Here, we compare five recently reported processes that produce Mito-EVs (Table 1) in depths.

**TABLE 1:** Comparison of five recently reported processes that produce Mito-EVs.

Research	Nicolás-Ávila et al., 2020	Tan et al., 2022	Liang et al., 2023	Jiao et al., 2021	Liu et al., 2023
Process name	Exophergenesis; Heterophagy	Autophagic secretion of mitochondria (ASM)	/	Mitocytosis	Mitopherogenesis
Mito-EV name	Exophers	/	/	Mitosomes (migrasomes)	Mitophers
EV size	3.5 ± 0.1 μm in diameter by imaging	Larger than 0.22 μm (filtration cut-off)	Large EVs 115–550 nm in diameter by nanoparticle tracking	Up to 3 μm in diameter (embedded Mito 240 nm) by imaging	720 nm in diameter (embedded Mito 600 nm) by imaging
EV markers	Externalized phosphatidylserine; LC3B	Not in CD63^+^ or CD9^+^ EVs	CD81, ALIX, TSG101, LC3A/B, and p62; Not in CD63^+^ EVs	TSPAN4; WGA staining	Externalized phosphatidylserine
Mito status	Damaged (aberrant morphology, loss of ΔΨm, and ↓citrate synthase activity)	Damaged (↑Ser65 p-Ub)	Intact	Damaged (aberrant morphology, loss of ΔΨm, and ↑ROS)	Healthy (normal morphology and normal ΔΨm)
Donor cell type	Cardiomyocytes	HeLa	MEFs; Cardiomyocytes and cardiac tissues	Cancer cells (L929 and MiaCaPa-2), normal cells (NRK and HUVEC), macrophages (BMDM and peritoneal), and neutrophils	*C. elegans* spermatids
Recipient cell type	Cardiac-resident macrophages (cMacs)	HeLa	Raw 264.7 macrophages and cMacs	/	/
Function or consequences	Maintain mitochondrial homeostasis in the heart; Prevent inflammasome activation and autophagic block	Maintain mitochondrial homeostasis in cells without mATG8- conjugation and activate cGAS- STING pathway in recipient cells	Maintain mitochondrial homeostasis in cells with defective lysosome and prevent inflammasome activation	Maintain mitochondrial homeostasis in migrating cells	Control quantity of mitochondria and maintain sperm motility and fertility
Activators	↑ Autophagy: rapamycin	↓ mATG8 lipidation: ATG7 Δ, ATG3 Δ, ATG5 Δ	↓ Lysosomal function: bafilomycin A1, chloroquine; RAB7A Δ, T22N mutation; LAMP2 Δ,	↑ Mito stress: Low dose of CCCP, DFP, antimycin A, and oligomycin	↑ Extracellular protease: SWM-1 (protease inhibitor) Δ, pronase
	↓ cMacs: short- term Δ	AND ↑ Mito stress: antimycin A / oligomycin, and exhaustive exercise	Danon Disease mutations; aging (heart)	↓ Serum: serum starvation	↑ Mito outward translocation: SPE- 15 (myosin VI) Δ or inhibitor 2,4,6- triiodophenol
	↑ Cardiac stress: isoproterenol	(RAB7A Δ increases basal but not damage- induced ASM)	↓ ΔΨm: FCCP	↑ Mito translocation: dynein Δ	↑ Actin polymerization: phalloidin
Inhibitors	↓ Autophagy: ATG7 Δ in donor cells	↓ Autophagosome biogenesis: ATG9 Δ; FIP200 Δ,	↓ EV release: GW4869	↓ Mito translocation: KIF5B Δ, Myo19 Δ	↓ Extracellular protease signaling: TRY-5 (protease) Δ, SPE-12
	↓ cMacs: long-term Δ	ULK1/2 inhibitor SBI-0206965;	↓ MVB-PM fusion: RAB27A Δ	↓ Mito fission: Drp1 Δ	(transmembrane protein) Δ, SPE-8 (tyrosine kinase) Δ
		ATG14 Δ, class III PI3K inhibitor SAR405		↓ Migrasome formation: TSPAN9 Δ, and myosin II inhibitor blebbistatin	↓ Actin polymerization: latrunculin A, CK- 636
		↓ Autophagosome- PM fusion: SNAP23 Δ			
The mechanisms of Mito-EV formation	Not determined	PINK1-Pakin and NDP52 mediate mitophagosome formation without mATG8 lipidation. SNAP23 mediates mitophagosome fusion to PM. Secreted Mito is likely non- enveloped	When lysosomal function is impaired, Mitos are sequestered in CD81^+^vesicles. RAB27A mediates Mito- EV secretion, likely through MVB-PM docking/fusion	Microtubule motor KIF5B and actin motor Myo19 mediate damaged Mito translocation to PM. Drp1 mediates Mito fission. The Mito fragments on retraction fibers move into migrasomes	One healthy Mito is exported to PM on actin filaments. Extracellular protease, SPE-12, and SPE-8 mediate the signaling. Mitopher is formed by PM budding
Autophagy proteins involved (donor cells)	LC3B, ATG7	ATG9A, ULK1 complex, and PI3KC3-C1 complex	Independent of mATG8 lipidation	Not tested	Not tested
		Independent of mATG8 lipidation	LC3A/B, p62 may also secrete in CD81^+^ EVs		
Lysosomal proteins involved	Recipient cells: LAMP1 (Mito-LAMP1 colocalization)	Donor cells: Independent of lysosomal degradation (↓Mito-LAMP2 colocalization; bafilomycin A1 insensitive)	Donor cells: LAMP2	Not tested	Not tested
			Recipient cells: lysosomal degradation after uptake		
Relationship with mitophagy	Not determined	ASM is independent of mATG8 lipidation and autophagosome- lysosome fusion, while dependent on secretory autophagy	High doses of mitophagy- inducer FCCP induce Mito-EV secretion, which is independent of functional mitophagy (ATG5, ATG7, PINK1)	Low doses of CCCP induce mitocytosis, while high doses of CCCP induce mitophagy	Mitophagy may not occur as spermatids lack lysosomes

Mito, mitochondria; EV, extracellular vesicle; ΔΨm, mitochondrial membrane potential; PM, plasma membrane; MVB, multivesicular body; ↑, increase; ↓, decrease; Δ, knockdown or knockout.

Mito-EVs vary in size and markers. Cardiac exophers are the largest, ranging from 3.5 ± 0.1 μm in diameter, positive for the “eat-me” signal externalized phosphatidylserine, and the autophagosomal marker LC3 (Nicolas-Avila et al., 2020), while lysosomal impairment-induced Mito-EVs are the smallest, ranging from 115 to 550 nm in diameter, positive for multivesicular body (MVB) and EV markers CD81, ALIX, and TSG101 but not CD63, as well as autophagosomal markers LC3 and SQSTM1/p62 (Liang et al., 2023). Mitosomes contain multiple small mitochondria of 240 nm size on average, positive for the migrasomal marker TSPAN4 (Jiao et al., 2021). During the autophagic secretion of mitochondria (ASM), the secreted mitochondria are bigger than 0.22 μm, negative for the EV markers CD63 and CD9 (Tan et al., 2022). The mitochondria in the abovementioned processes are mostly damaged, assessed by morphology, mitochondrial membrane potential (ΔΨm), or other markers (Nicolas-Avila et al., 2020; Jiao et al., 2021; Tan et al., 2022). In contrast, mitophers are 720 nm in diameter, positive for externalized phosphatidylserine, embedding a single healthy mitochondrion of 600 nm size on average with normal morphology and ΔΨm (Liu et al., 2023).

Mito-EVs function in the damaged mitochondria quality control or healthy mitochondria quantity control. Cardiac exophers and lysosomal impairment-induced CD81-positive Mito-EVs can be secreted from cardiomyocytes and taken up by macrophages in the neighborhood for degradation to prevent inflammation (Nicolas-Avila et al., 2020; Liang et al., 2023), a process called heterophagy (Nicolas-Avila et al., 2022). Cardiac-resident macrophages (cMacs) play an important role in maintaining mitochondrial homeostasis in the heart: short-term depletion of cMacs results in an accumulation of cardiac exophers, while the long-term depletion of cMacs reduces the formation of cardiac exophers through inflammasome activation and autophagy block in cardiomyocytes (Nicolas-Avila et al., 2020). ASM takes place upon both mATG8-lipidation deficiency and mitochondrial stress in donor cells and stimulates cGAS–STING-mediated innate immune response in recipient cells of the same type (Tan et al., 2022). Mitosomes are a kind of migrasomes, released by various migrating cells upon low doses of mitochondrial stressors (Jiao et al., 2021). Mitophers bud off *C. elegans* spermatids to reduce around 1/3 quantity of mitochondria to maintain sperm motility and fertility (Liu et al., 2023). It is not clear how mitosomes and mitophers are ultimately cleared; we speculate that they can be internalized by other cells, for elimination or for material and/or signal transfer, since several kinds of migrasomes have been implicated to regulate lateral transfer of cellular contents (Yu and Yu, 2022; Zhang et al., 2023) and worm exophers produced from body wall muscles, a small population of which contains intact mitochondria and can transport yolk proteins to oocytes (Turek et al., 2021).

The molecular mechanisms underlying the formation of Mito-EVs are characterized at different stages, including upstream signaling transduction, mitochondria translocation, cargo recognition, vesicle fusion, or budding. Mitophers are triggered by a signaling cascade consisting of extracellular protease TRY-5, transmembrane protein SPE-12, and tyrosine kinase SPE-8 (Liu et al., 2023). Microtubules, actin cytoskeleton, and motor proteins play a vital role in mitochondria translocation. Actin polymerization is required and is sufficient to promote mitopher formation. Myosin VI SPE-15 negatively regulates mitochondrial outward transport on actin filaments (Liu et al., 2023). During mitocytosis, the microtubule outward motor KIF5B and the actin motor Myo19 facilitate the transport of tubular mitochondria to the bottom of the cells to form mitosomes, while microtubule inward motor dynein inhibits the process; the tips of tubular mitochondria undergo Drp1-mediated fission before the mitochondrial fragments on the retraction fibers are packed into migrasomes, whose formation requires cell migration and tetraspanin proteins (Jiao et al., 2021). Autophagy proteins exhibit complex functions in cargo recognition and vesicle formation during cardiac exophergenesis, ASM, and lysosomal impairment-induced mitochondrial secretion, which are discussed in details in the later section. To release Mito-EVs, RAB27A mediates the docking and fusion of MVB to the plasma membrane (Liang et al., 2023), whereas SNAP23 mediates the fusion of mitophagosome to the plasma membrane, and the authors suggest that the secreted mitochondria are non-enveloped during ASM (Tan et al., 2022). Mitophers are released by direct budding from the plasma membrane, although the molecular mechanism remains elusive (Liu et al., 2023). It is not examined how cardiac exophers get released (Nicolas-Avila et al., 2020). Since cardiac exophers contain mitophagosomes, a fusion step between MVB or amphisomes to the plasma membrane may occur.

### Roles of autophagic and lysosomal pathways on Mito-EVs

Mito-EVs show different dependencies on the autophagic pathway. Cardiac exophers, positive for LC3, require the mATG8-conjugation system as ATG7 knockdown in cardiomyocytes reduces the number of cardiac exophers, and autophagy induction by rapamycin is sufficient to induce cardiac exopher production (Nicolas-Avila et al., 2020). The mATG8-conjugation system is dispensable for lysosomal impairment-induced CD81-positive Mito-EV secretion since ATG5 or ATG7 knockdown shows no effect; interestingly, LC3 and p62 are secreted in CD81-positive EVs upon the same treatment (Liang et al., 2023), suggesting that Mito-EVs may carry such cargos as ubiquitinated proteins, and LC3/p62-dependent and independent EV cargo sorting pathways may co-exist. Consistent with this thought, LIR-containing RNA-binding proteins have been reported to secrete in LC3-positive EVs through a process named LC3-dependent EV loading and secretion (LDELS) (Leidal et al., 2020). When the mATG8-conjugation system is defective (ATG3-, ATG5-, and ATG7-knockout), PINK1–Parkin and the mitophagy cargo receptor NDP52 mediate the sorting of damaged mitochondria; upstream ATG proteins such as ATG9A, ULK1 complex, and PI3KC3-C1 complex control the formation of mitophagosomes, which later fuse with the plasma membrane instead of lysosomes (Tan et al., 2022). These results indicate an inhibitory role of mATG8-conjugation machinery and a positive role of upstream ATG proteins on secretory autophagy. Notably, not all studies have evaluated ATG proteins upstream of mATG8 conjugation, and it remains elusive whether ATG proteins mediating autophagosome initiation and elongation regulate the formation of CD81-positive Mito-EVs, mitosomes, and mitophers, although the authors claim they are autophagy-independent.

Mito-EV formation barely needs lysosomal function. Worm spermatids that generate mitophers lack lysosomes (Liu et al., 2023). When lysosomal function is compromised by pharmacological inhibitors (bafilomycin A1 and chloroquine), RAB7A depletion or dysregulation (inactive mutation T22N), LAMP2 depletion or Danon disease-causing mutations, or aging, CD81-positive Mito-EVs take charge to remove mitochondria (Liang et al., 2023). Pharmacological inhibition of lysosomes does not affect ASM, and the colocalization of mitochondria and LAMP2 is reduced during ASM (Tan et al., 2022). It is worth noting that after Mito-EVs are captured by recipient cells, especially macrophages, the clearance of Mito-EVs still involves lysosomal degradation (Nicolas-Avila et al., 2020; Liang et al., 2023). This suggests that when mitophagy cannot occur in donor cells, Mito-EV secretion and heterophagy in recipient cells are compensatory processes.

### Dual roles of secretory autophagy

In the past decade, an accumulating body of evidence has uncovered the role of secretory autophagy in conventional and unconventional secretions of a wide range of cargos for cellular homeostasis or intercellular communications (Ponpuak et al., 2015; Piletic et al., 2023). However, the definition of secretory autophagy is still vague. For a cargo secretion process to be considered secretory autophagy, how many autophagy proteins, which may function at different stages of the autophagic pathway, shall be required? Shall autophagy proteins be on or in the cargo carriers? Shall autophagy proteins be sufficient to promote cargo secretion upon activation? It is urgent for the field to reach a consensus. Furthermore, the relationship between degradative and secretory autophagy needs in-depth investigation. It is intuitive to assume that the divergence point of degradative and secretory autophagy is the fusion step of autophagosomes. When autophagosome fuses with lysosome, cargos undergo degradative autophagy; when autophagosome fuses with late endosome, MVB, and/or the plasma membrane, cargos undergo secretory autophagy. However, as discussed above, neither ASM (Tan et al., 2022) nor lysosomal impairment-induced CD81-positive Mito-EV secretion (Liang et al., 2023) requires the mATG8-conjugation system, suggesting that the divergence point of degradative and secretory autophagy is rather upstream, likely the formation step of autophagosomes.

**FIGURE 1:**
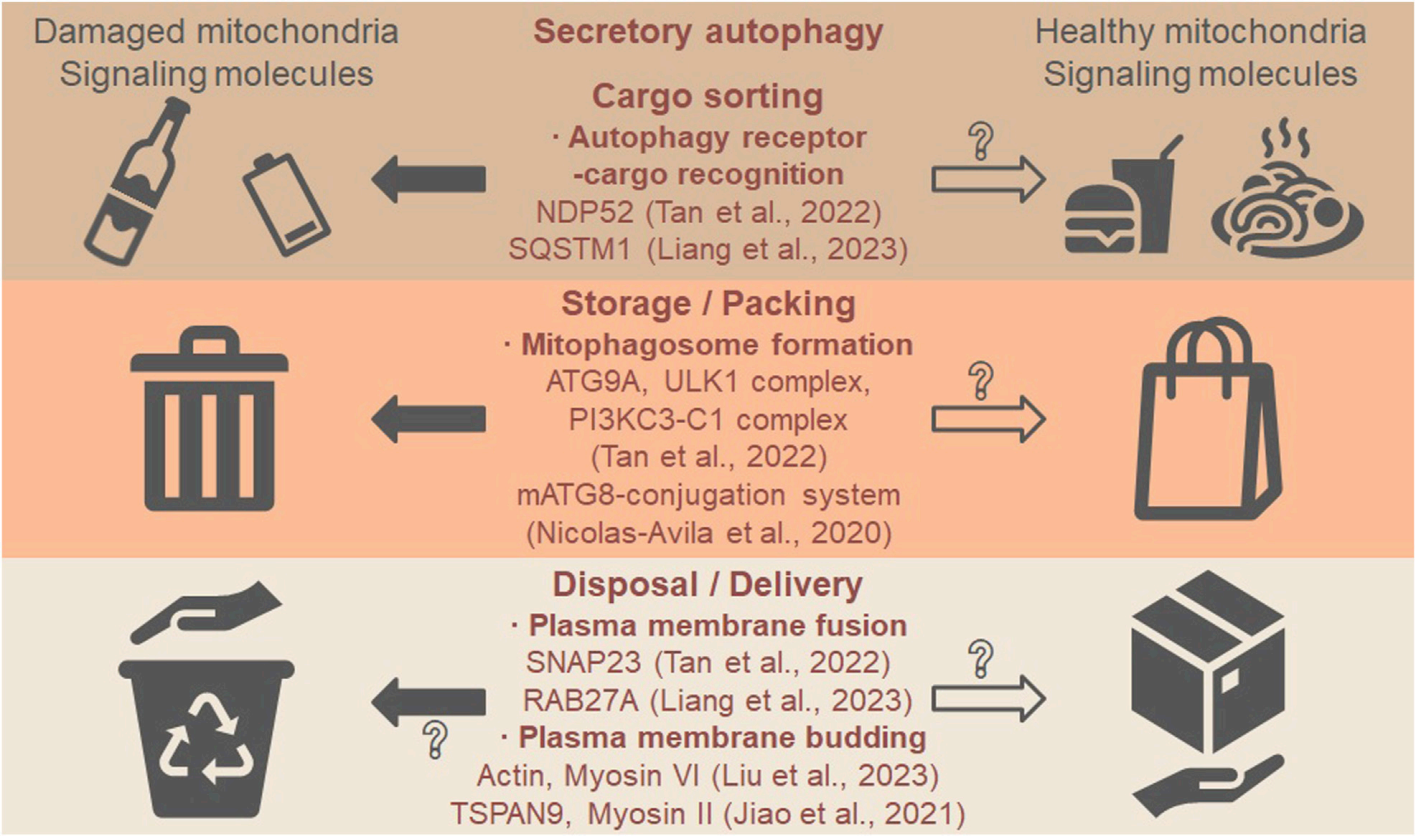
Secretory autophagy may mediate the secretion of mitochondria and signaling molecules. The first step is cargo sorting where autophagy receptors recognize such cargos as damaged mitochondria (“waste”) and healthy mitochondria (“food”). The second step is the formation of mitophagosomes, resembling waste storage and food packing. The last step is plasma membrane fusion or budding, resembling waste disposal and food delivery. In the five recent studies on Mito-EVs discussed in this opinion, the autophagy receptor NDP52 regulates the sorting of damaged mitochondria (Tan et al., 2022), and the autophagy receptor SQSTM1/p62 regulates the sorting of ubiquitinated proteins to CD81-positive Mito-EVs (Liang et al., 2023). ATG9A, ULK1 complex, and PI3KC3-C1 complex control the formation of mitophagosomes (autophagosomes that contain mitochondria) (Tan et al., 2022), while the mATG8-conjugation system may also be required (Nicolas-Avila et al., 2020). The fusion to the plasma membrane is mediated by SNAP23 for mitophagosomes (Tan et al., 2022) or by RAB27A for MVB containing CD81-positive Mito-EVs (Liang et al., 2023). The direct budding from the plasma membrane is regulated by actin cytoskeleton and myosin VI motor to form mitophers (each contains a healthy mitochondrion) (Liu et al., 2023) or mediated by the tetraspanin protein TSPAN9 and myosin II motor to form mitosomes (specialized migrasomes) (Jiao et al., 2021), although it is not clear if direct budding is involved in secretory autophagy. Whether healthy mitochondria are sorted, packed, and delivered via similar mechanisms require further investigation (open arrows and question marks).

Mitochondria are well-known cargos of degradative autophagy (see review (Picca et al., 2023)), and they could be cargos of secretory autophagy as well (Table 1). The secretion of cardiac exophers sets a good example of secretory autophagy because (1) autophagy is necessary for the Mito-EV secretion (ATG7-dependent); (2) autophagy protein is on the Mito-EV (LC3-positive); and (3) autophagy is sufficient for the Mito-EV secretion (rapamycin-induced). ASM shares the first two features with the secretion of cardiac exophers, the necessity of autophagy (ATG9A, ULK1 complex, and PI3KC3-C1 complex-dependent) and the presence of autophagy protein in the carriers (NDP52-positive), while the sufficiency of autophagy has not been tested. We propose that the following steps are involved in the secretory autophagy of mitochondria, applicable to different types of Mito-EVs: cargo sorting *via* autophagy receptor-cargo recognition, autophagosome formation, and the plasma membrane fusion or budding (Figure 1). Future studies shall evaluate more ATG proteins than the mATG8-conjugation system to determine whether secretory autophagy occurs.

In addition, we propose that both damaged and healthy mitochondria could be the cargos of secretory autophagy (Figure 1, “waste” vs. “food”): different cargos are sorted *via* distinct receptor–cargo pairs and packed into mitophagosomes. EVs that contain healthy mitochondria can be transferred from bone marrow-derived stromal cells to alveolar epithelial cells in injured lungs, restoring bioenergetics and improving survival (Islam et al., 2012). Healthy mitochondria-containing EVs can also be isolated from stem cell-derived cardiomyocytes, taken up by cardiomyocytes in failing hearts, restoring bioenergetics and improving cardiac function (Ikeda et al., 2021). Although the role of secretory autophagy in healthy mitochondria release has not been reported, we think worm exophers which are produced from body wall muscles and delivered to oocytes may be the case. Muscular exophers contain organelles like intact mitochondria and large protein complexes, and the production is autophagy-dependent (ATG7 and ATG8-dependent) (Turek et al., 2021). The secretory autophagosomes may encapsulate various cargos and signaling molecules, which is common for degradative autophagosomes. After fusing with or budding off the plasma membrane, the cargos can be disposed to the extracellular space or delivered to recipient cells. Future studies shall focus on the cargo sorting mechanisms of mitochondria and identify additional cargos on the same ride.

In summary, Mito-EVs are novel structures that cells generate for mitochondrial quality or quantity control, some of which rely on secretory autophagy. Future research on autophagy proteins and Mito-EVs will not only shed light on the role of secretory autophagy in mitochondrial homeostasis but also on the mechanistic distinctions between degradative and secretory autophagy.
